# Detailed Analyses of the Expression Patterns of Potential Severe Acute Respiratory Syndrome Coronavirus 2 Receptors in the Human Heart Using Single-Nucleus RNA Sequencing

**DOI:** 10.3389/fcvm.2021.757362

**Published:** 2021-11-30

**Authors:** Jie Ren, Yuze Zhang, Shishi Liu, Xiangjie Li, Xiaogang Sun

**Affiliations:** ^1^Fuwai Hospital, National Center for Cardiovascular Diseases, Chinese Academy of Medical Sciences and Peking Union Medical College, Beijing, China; ^2^School of Statistics, Renmin University of China, Beijing, China; ^3^School of Statistics and Data Science, Nankai University, Tianjin, China

**Keywords:** SARS-CoV-2, cardiac injury, cellular receptors, ACE2, expression features

## Abstract

Cardiac injury is a common complication of coronavirus disease 2019 (COVID-19), but the exact mechanisms have not been completely elucidated. The virus receptors on subsets of cells are key determinants of susceptibility to severe acute respiratory syndrome coronavirus 2 (SARS-CoV-2) infection. Due to its high sequence similarity to SARS-CoV, SARS-CoV-2 also utilizes ACE2 as the cell entry receptor. A growing number of studies have indicated that other receptors apart from ACE2 are involved in SARS-CoV-2 infection. This study aimed to elucidate the expression characteristics of SARS-CoV-2 cellular receptors in the heart. We first investigated ACE2 expression in a comprehensive transcriptional landscape of the human heart comprising single-nucleus RNA-seq (snRNA-seq) data for >280,000 cells. Then, the expression distributions of novel SARS-CoV-2 receptors were analyzed at the single-cell level to clarify the cardiovascular complications in COVID-19. We observed a higher percentage of ACE2-positive cells in pericytes (8.3%), fibroblasts (5.1%), and adipocytes (4.4%) in the human heart, compared to other cell types. The frequency of ACE2-positive cells in each cell type from the ventricles was significantly higher than that in the atria, suggesting that the ventricular cells are more susceptible to SARS-CoV-2 infection. The distribution patterns of other receptors (BSG, HSPA5, KREMEN1, NRP1, ANPEP, AXL) were significantly different from those of ACE2, demonstrating higher expression levels in ventricular cardiomyocytes. Moreover, our results suggest that fibroblasts and adipocytes, aside from pericytes, may be vulnerable targets for SARS-CoV-2 infection in the human heart. Our study presents potential targets for future clinical studies and interventions for cardiac injury in patients with COVID-19.

## Introduction

The coronavirus disease 2019 (COVID-19), caused by a pathogenic coronavirus known as severe acute respiratory syndrome coronavirus 2 (SARS-CoV-2) (1), has become a serious global public health issue (2). Currently, there are no specific therapeutics available for the treatment of COVID-19 (3). Respiratory system symptoms were the predominant clinical manifestations in patients with COVID-19, similar to severe acute respiratory syndrome (SARS) and the Middle East respiratory syndrome (4). However, patients with COVID-19 seem to have a diverse range of extrapulmonary manifestations (5–8). Cardiac injury is a common complication of COVID-19 and is observed in 7–20% of patients infected with SARS-CoV-2 (9). Moreover, SARS-CoV-2 infection in the myocardium was verified by reverse transcription polymerase chain reaction (RT-qPCR) and histopathology (8, 10). The presence of myocardial injury is an independent risk factor for mortality in patients with COVID-19 (11). However, the exact mechanisms underlying cardiac injury in patients with COVID-19 have not been completely elucidated.

As essential elements of virus transmission, the expression and distribution of virus receptors on subsets of cells are key determinants of susceptibility to SARS-CoV-2 infection (12). SARS-CoV-2 has ~79.6% genome sequence homology with SARS-CoV (13, 14). Thus, angiotensin-converting enzyme 2 (ACE2) is thought to play an important role in the attachment and entry of SARS-CoV-2 and the pathogenesis of COVID-19 (4, 12, 15). Cells expressing ACE2 may be considered as a therapeutic target for COVID-19 (16). Some studies have demonstrated that ACE2 is highly expressed in pericytes in the human heart (17), but it has not been well-studied because of the lack of a comprehensive human single-cell atlas. It is of great significance to gain deeper insights into the expression characteristics of SARS-CoV-2 cellular receptors in the heart.

It is worth noting that certain organs with little ACE2 expression was also detected for virus invasion (18). The different clinical features of COVID-19 and SARS (19) also demonstrated that there may be other receptors and/or co-receptors of SARS-CoV-2, which are involved in the entry of the virus into cells (20). High-throughput receptor profiling for SARS-CoV-2 revealed that the S protein could specifically bind to the following cellular receptors with high affinity: ASGR1, KREMEN1 (21), ADAM17, GRP78, and CD147 (22). Recent studies have also suggested the possible involvement of NRP1 (23), ANPEP (20, 24), and AXL (25) in SARS-CoV-2 attachment, subsequent entry into the cell, and infection. Therefore, the expression features of the recently discovered SARS-CoV-2 receptors in human cardiac cells should be explored.

In this study, we first investigated the ACE2 expression in a comprehensive transcriptional landscape of the human heart comprising single-nucleus RNA sequencing (snRNA-seq) data for >280,000 cells (26). Then, the expression distributions of the novel SARS-CoV-2 receptors were also analyzed at the single-cell level to advance our understanding of cardiovascular complications in COVID-19.

## Methods

### Public Dataset Acquisition

We acquired the available snRNA-seq data derived from seven normal human heart samples from the Broad Institute's Single Cell Portal^1^ under the Study ID SCP498 online. In which, “healthy_human_4chamber_map_unnormalized_V3.h5ad” was used in the downstream analyses. We used the gene expression matrix and cell type annotation provided by the original article (26).

### Processing and Visualization of snRNA-Seq Data

Since the low-quality cells were excluded from the original publication, we used all the data available. The downloaded unique molecular identifier count matrix was converted to Seurat object using the R package Seurat v.3.2.2 (27). We then normalized the raw gene expression matrix using the NormalizeData function and visualize the expression level using the Violin plot function in Seurat. Uniform manifold approximation and projection (UMAP)^2^ was used to visualize the results.

Downstream analyses, including highly variable gene detection (FindVariableFeatures, method= “vst,” nfeatures = 2,000), data feature scaling (ScaleData), PCA (Principal Component Analysis, RunPCA, from highly variable genes), an integration procedure (RunHarmony, npcs = 23), neighbor construction (FindNeighbors, using the output of RunHarmony), and clustering (FindClusters, resolution = 0.4), were performed using the R package Seurat (v.3.2.2) and harmony (v1.0). Cell type labels were assigned manually using the annotation provided in the original article (26). We further investigated clusters for which the genes indicated additional diversity and specificity.

### Differential Expression Analysis

Unless otherwise stated, tests for differentially expressed genes (DEGs) were performed using the FindAllMarker or FindMarker function in the Seurat package (with the default test method: Wilcoxon rank-sum test). To compute for the DEGs, all genes were included and expressed in at least 25% of cells in either of the two populations compared.

### Cell–Cell Interaction

Cell–cell interaction weights for each ligand-receptor pair were computed as the product of the fold change of ligands in sender-cell types and the fold change of the corresponding receptors in receiver cell types. We used the “layout_nicely” function in the R package “igraph” to visualize the cell-cell interaction.

### Software Availability

The R and Python scripts used to analyze the data in this study are available from the corresponding authors upon reasonable request (Table 1).

**Table 1:** Software and algorithms.

**Software (version)**	**Source**	**Link**
clusterProfiler v3.10.1	R package	https://github.com/YuLab-SMU/clusterProfiler
ComplexHeatmap v2.1.2	R package	https://github.com/jokergoo/ComplexHeatmap
cowplot v1.0.0	R package	https://github.com/wilkelab/cowplot
dendextend v1.13.4	R package	https://github.com/talgalili/dendextend
ggplot2 v3.2.0	R package	https://github.com/tidyverse/ggplot2
igraph v1.2.5	R package	https://igraph.org/r/ https://github.com/igraph/igraph
openxlsx v4.1.4	R package	https://github.com/awalker89/openxlsx
org.Hs.eg.db v3.8.2	R package	https://bioconductor.org/packages/release/data/annotation/html/org.Mm.eg.db.html
Parallel v3.6.1	R package	https://github.com/reborg/parallel
Pheatmap v1.0.12	R package	https://github.com/raivokolde/pheatmap
reticulate v1.15	R package	https://github.com/rstudio/reticulate
scanpy v1.4.6	Python package	https://github.com/theislab/scanpy/ https://scanpy-tutorials.readthedocs.io/en/latest/#
Seurat v3.2.2	R package	https://satijalab.org/seurat/ https://github.com/satijalab/seurat
Harmony v1.0	R package	https://github.com/immunogenomics/harmony

### Statistical Analysis

All statistical tests were performed using the R statistical programming language (V.3.6.2, R Foundation for Statistical Computing, Vienna, Austria). For analysis of snRNA-seq data, we used Wilcoxon rank-sum tests (between two groups) or Kruskal–Wallis test (for comparisons of multiple groups) to detect differential genes, with *P*-values adjusted based on Bonferroni correction. Statistical significance was set at *P* < 0.05.

## Results

### ACE2 Expression in the Human Heart

**Figure 1:**
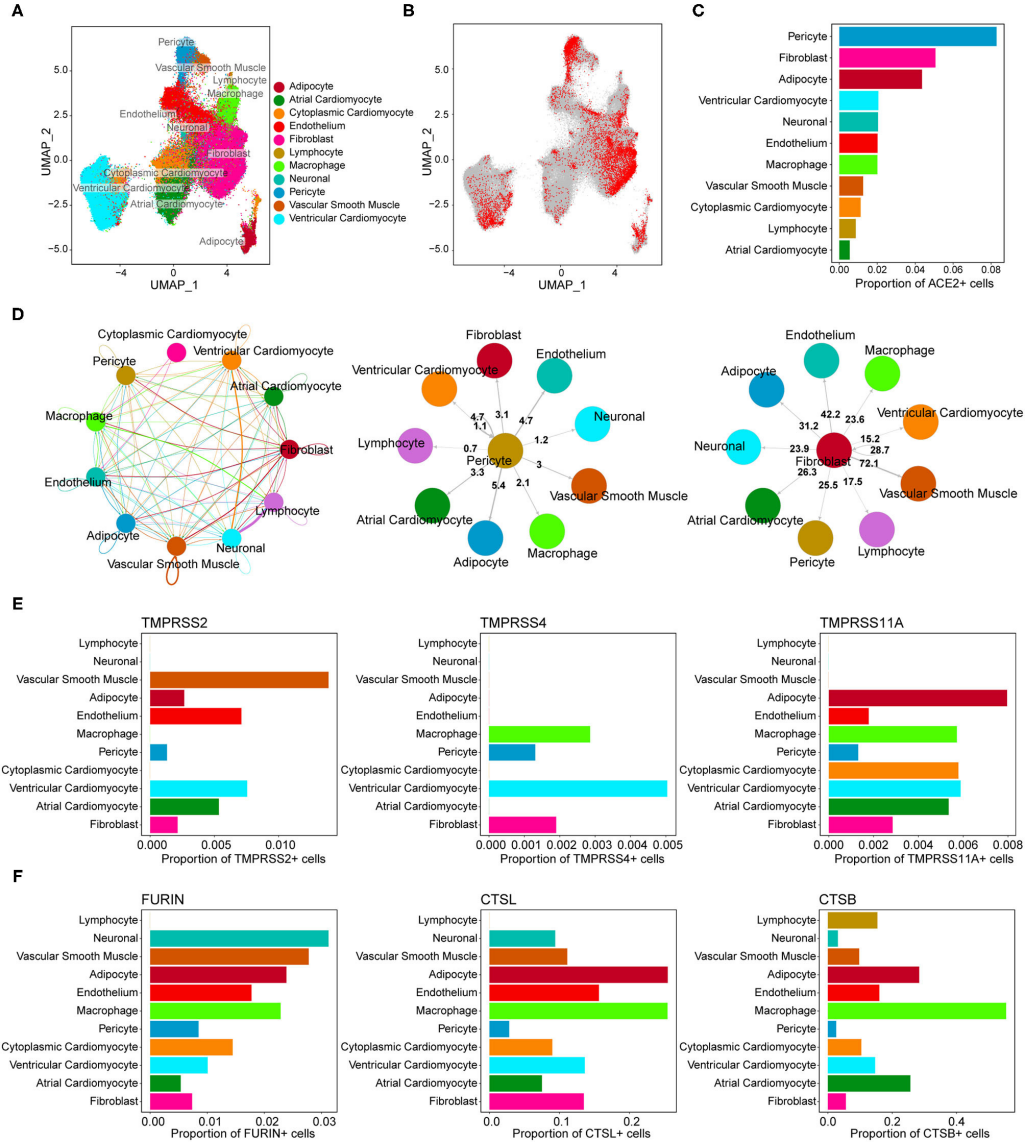
Gene expression characterizations of angiotensin-converting enzyme 2 (ACE2) and its co-receptors in the human heart. **(A)** Uniform manifold approximation and projection (UMAP) plot of cell clusters in the human heart. **(B)** Feature plot of ACE2 expression across all cell clusters. **(C)** The proportion of ACE2-positive cells in each cell population. **(D)** Reciprocal interaction network between different cell types. (Middle panel) Cell-cell interaction between pericyte (ligand) and other cell types (receptors). (Right panel) Cross-talks from fibroblast to other cell types. **(E,F)** The co-expression percentage of ACE2 and accessory proteases, such as TMPRSS2, FURIN, CTSL, and CTSB.

We obtained single-nucleus transcriptome data of seven human donor hearts containing 287,269 nuclei sequenced by 10X Genomics from the previous study (26). The basic characteristics of the human donors are shown in Supplementary Table 1. As shown in Figure 1A, unsupervised graph-based clustering revealed 11 cell types in the human heart, of which multiple cell types expressed the ACE2 gene (Figure 1B). Clear differences in the proportion of ACE2-positive cells were found among the different cell types (Figure 1C). Furthermore, high percentages of ACE2-positive cells were observed in the pericytes (8.3%), fibroblasts (5.1%), and adipocytes (4.4%) in the heart (Table 2). These results are slightly different from those reported in the literature (17). In addition to pericytes, we predicted that fibroblasts and adipocytes could be vulnerable targets for SARS-CoV-2 infection. Similar to previous reports, the pericyte showed a significant intercellular interaction with the endothelium (Figure 1D). In addition, fibroblast demonstrated significant reciprocal interactions with vascular smooth muscle cells (Figure 1D). These results suggest that vascular injury plays an important role in the occurrence and development of cardiac injury in COVID-19. After binding to the cell surface, SARS-CoV-2 requires S protein cleavage proteases, such as TMPRSS2, FURIN, CTSL, and CTSB, to facilitate cell entry by inducing the fusion of cellular and viral membranes. TMPRSS2/4/11A, the well-known co-receptor for SARS-CoV-2 in other organs, demonstrated almost no co-expression (<1%) with ACE2 in various cell types of the human heart (Figure 1E; Supplementary Table 2). However, FURIN and CTSL/B seemed to play significant roles as co-receptors for SARS-CoV-2 entry into the human heart (Figure 1F). Further studies are required to elucidate the exact mechanism.

**Table 2:** The expression features of angiotensin-converting enzyme 2 (ACE2) in the human heart.

**Cell type**	**Num_of_**	**Num_of_**	**Prop_of_**
	**cells**	**ACE2_cells**	**ACE2_cells**
Fibroblast	83,220	4,219	0.050696948
Atrial cardiomyocyte	34,051	187	0.005491762
Ventricular cardiomyocyte	57,906	1,191	0.020567817
Cytoplasmic cardiomyocyte	30,765	346	0.011246546
Pericyte	18,467	1,531	0.082904641
Macrophage	17,468	350	0.020036638
Endothelium	27,923	563	0.02016259
Adipocyte	8,658	377	0.043543544
Vascular smooth muscle	5,740	72	0.012543554
Neuronal	1,568	32	0.020408163
Lymphocyte	1,503	13	0.008649368

**Figure 2:**
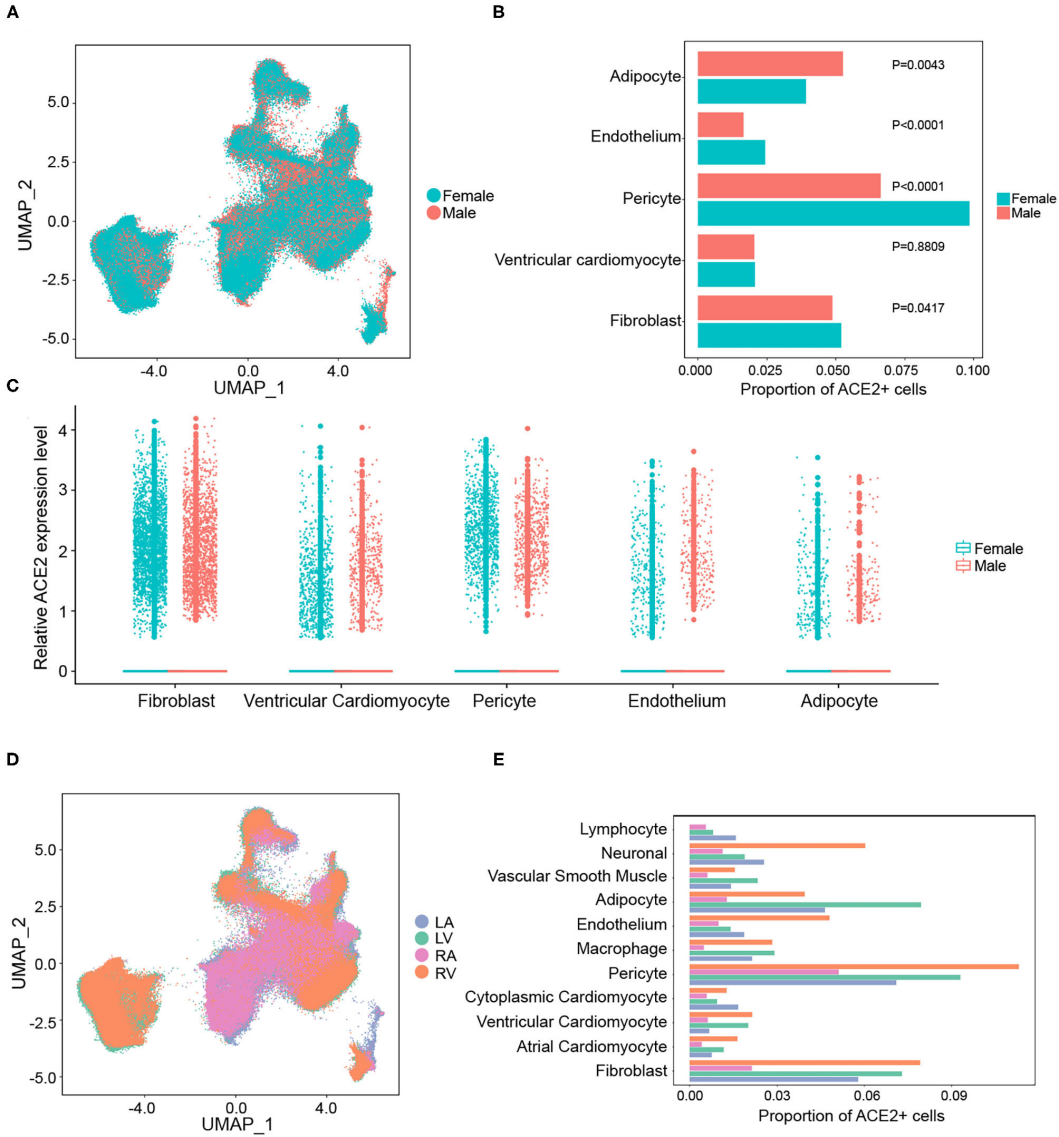
Gender and regional differences in the expression of ACE2 in the human heart. **(A)** The overall distribution of cardiac cells between sexes. **(B)** The gender-differentiated effect on the percentage of ACE2-positive cells in main cell clusters. **(C)** The expression levels of ACE2 between sexes in different cell types. **(D)** The distributions of gene expression in cells from different chambers of the heart. **(E)** The proportion of ACE2-positive cells in each cell cluster from different cardiac chambers.

It has been proven that the male sex is a risk factor for disease severity in COVID-19 (28). The heart is composed of different chambers, which have distinct structural and functional characteristics. Hence, gaining a deeper understanding of the expression characteristics of SARS-CoV-2 cellular receptors in the human heart at spatial and sex-specific levels is of great importance. No significant differences in the overall distribution of cardiac cells between the sexes were observed (Figure 2A). The proportion of ACE2-positive cells was further compared between different genders in each cell type. As shown in Figure 2B, the ratio of ACE2-positive cells had a gender-differentiated effect in pericytes, fibroblasts, and the endothelium. However, it is interesting to note that even with a lower ACE2 proportion, higher expression levels of ACE2 were observed in pericytes, fibroblasts, and endothelium in male hearts (Figure 2C). This might be a reason why there is no significant difference observed in adverse cardiac events between patients with COVID-19 of different genders.

However, there were remarkable differences in the gene expression between the atria and ventricles of the human heart (Figure 2D). The frequency of ACE2-positive cells in each cell type from the ventricles was significantly higher than that in the atria (Figure 2E). For instance, the proportion of ACE2-positive cells in ventricular cardiomyocytes (2.1%) were significantly higher than that in the atrial cardiomyocytes (0.5%). This suggests that human ventricular cells are more susceptible to SARS-CoV-2 infection.

### Expression Signatures of Potential SARS-CoV-2 Receptors in the Human Heart

**Figure 3:**
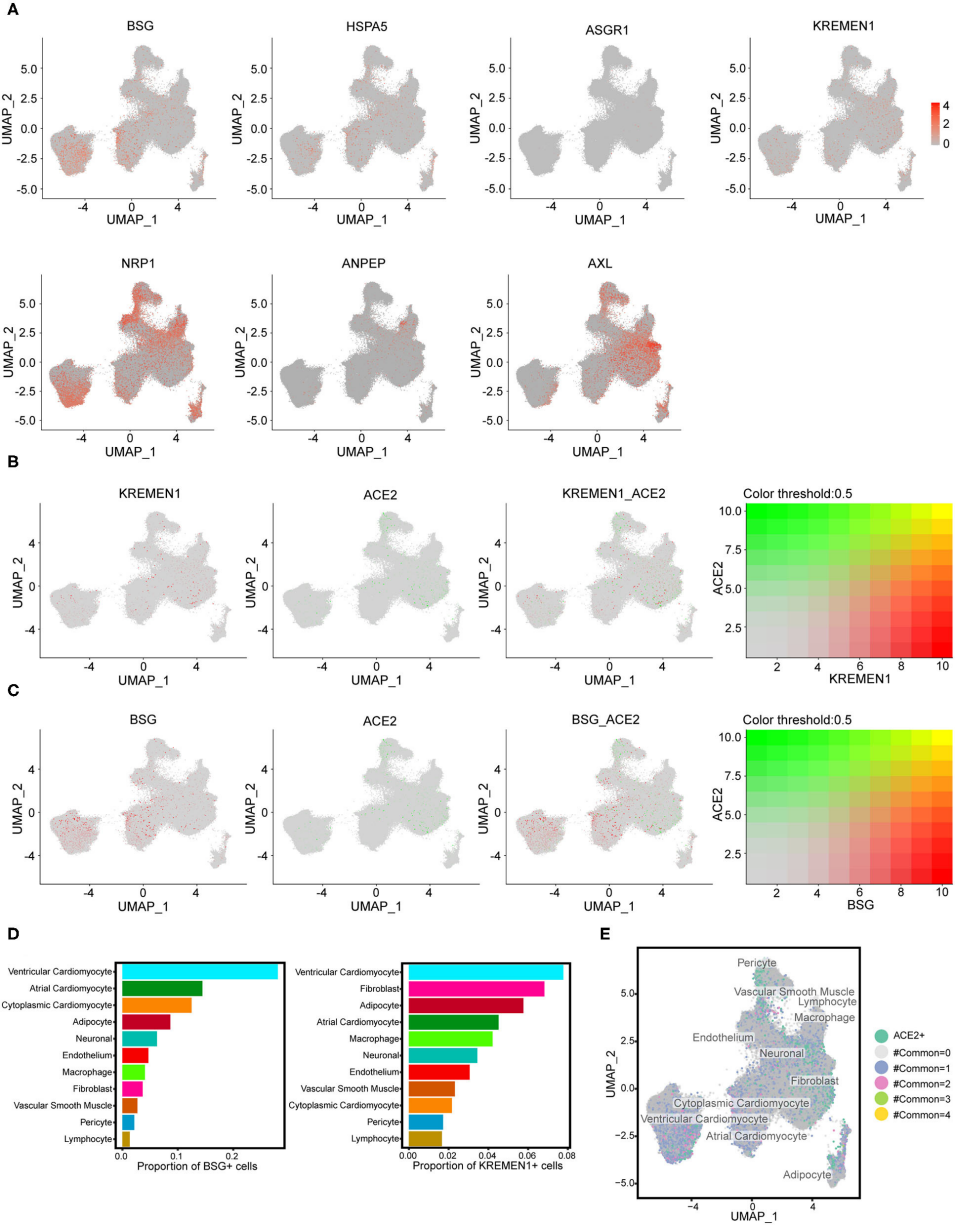
The expression signatures of potential receptors in the human heart. **(A)** Expression features of potential SARS-CoV-2 receptors (BSG, HSPA5, ASGR1, KREMEN1, NRP1, ANPEP, and AXL) across all cell clusters. **(B)** The expression distributions of KREMEN1 and its correlation with ACE2. **(C)** The expression distributions of BSG and its correlations with ACE2. For **(B,C)** right panels: green represents BSG/KREMEN1 positive cells, red represents ACE2 positive, gray indicates double-negative cells, and yellow indicates double-positive cells. **(D)** The proportion of BSG-positive or KREMEN1-positive cells in each cell population. **(E)** The expression characterizations of the four known receptors (ACE2, BSG, HSPA5, and KREMEN1). The numbers represent the receptors with a coexistence state.

Previous studies have reported potential receptors involved in SARS-CoV-2 binding and infection other than the ACE2 (20–25). Each potential receptor exhibited a distinct expression pattern (Figure 3A). The human cardiac cells showed nearly no expression of ASGR1, while the other receptors (BSG, HSPA5, KREMEN1, NRP1, ANPEP, and AXL) showed scattered expression in each cell type (Figure 3A; Supplementary Tables 3–5). The expression distributions and correlations of the potential receptors and ACE2 were analyzed using feature plots. There was no significant association between the distribution of ACE2 and the potential receptors (Figures 3B,C). Dissimilarities among the expression characteristics of the potential receptors, compared with ACE2, might be the reason for the different clinical features of COVID-19 and SARS. Significantly different from the distribution pattern of ACE2, positive expression of BSG, KREMEN1, and NRP1 were highest in the ventricular cardiomyocytes (Figures 3D,E). These results may shed light on the susceptibility of human ventricular cardiomyocytes to SARS-CoV-2.

## Discussion

The heart is one of the involved organs in COVID-19 (29). Using the results from the detailed expression patterns of ACE2 and the other potential receptors of SARS-CoV-2 in the human heart, we suggested that fibroblasts and adipocytes could serve as vulnerable targets for SARS-CoV-2 other than pericytes (17, 30). Moreover, human ventricular cells are more susceptible to SARS-CoV-2. Our study provides more information on the infectious mechanism of SARS-CoV-2 in the human heart.

Cardiac fibroblasts play important roles in normal physiology and pathological states, such as fibrosis (31). Fibroblasts, a major cell type in the heart, support the structural framework of tissue and maintain tissue homeostasis (32). Impaired consecutive fibroblasts can cause progressive cardiac dysfunction and increase the risk of sudden death (33, 34). Meanwhile, an imbalance or dysfunction of adipocytes could lead to inflammatory cascade reactions by secreting proinflammatory mediators (adipokines, IL-1, TNF-α, etc.) (35, 36), and these inflammatory markers could further play a role in the activation of immune cells (37). Epidemiological investigations have also demonstrated that obesity is a risk factor for a poor prognosis of COVID-19 (38). These observations suggest that fibroblasts and adipocytes are additional vulnerable targets for SARS-CoV-2. In addition, the mammalian atria and ventricles display striking differences in their structural features and gene expression (39). More than 2,000 genes appear to be differentially expressed in human ventricles and atria (26). The results of our study suggest that ventricular cells, especially cardiomyocytes, fibroblasts, adipocytes, and pericytes, are more likely to suffer from SARS-CoV-2 infection.

Recently, various investigations have suggested the existence of other receptors involved in SARS-CoV-2 infection, in addition to ACE2 (20–25). In this study, these receptors had relatively scattered distributions than ACE2 in the human heart. It is noteworthy that the expression levels of these receptors were significantly higher in ventricular cardiomyocytes, suggesting higher susceptibility of ventricular cardiomyocytes to SARS-CoV-2 infection. These potential SARS-CoV-2 receptors may play an important role in promoting viral infection of the human cardiac system and may be potential targets for future clinical cardiac intervention strategies.

### Limitations

First, our results and conclusions are data-driven, which needed further laboratory verifications. Additionally, the original sample size was relatively small, which restricted its further implications. Finally, the snRNA-seq data we included was from normal human hearts, which could, therefore, limit further analysis of the correlation between gene expression features and clinical characteristics in patients with COVID-19.

## Data Availability Statement

Publicly available datasets were analyzed in this study. This data can be found here: Broad Institute's Single Cell Portal (https://singlecell.broadinstitute.org/single_cell/study/SCP498/transcriptional-and-cellulardiversity-of-the-human-heart) under study ID SCP498.

## Author Contributions

XS contributed to the study's conception and design. Material preparation and data collection were performed by JR, YZ, and XL. SL and XL analyzed the data. The first draft of the manuscript was written by JR. All the authors commented on previous versions of the manuscript. All the authors read and approved the final manuscript.

## Funding

This study was supported by the Beijing Municipal Science and Technology Commission (Z181100001718197).

## Conflict of Interest

The authors declare that the research was conducted in the absence of any commercial or financial relationships that could be construed as a potential conflict of interest.

## Publisher's Note

All claims expressed in this article are solely those of the authors and do not necessarily represent those of their affiliated organizations, or those of the publisher, the editors and the reviewers. Any product that may be evaluated in this article, or claim that may be made by its manufacturer, is not guaranteed or endorsed by the publisher.

## Footnotes

^1^https://singlecell.broadinstitute.org/single_cell/study/SCP498/transcriptional-and-cellulardiversity-of-the-human-heart.

^2^https://github.com/lmcinnes/umap.
