# Supplementary material for: Detailed Analyses of the Expression Patterns of Potential Severe Acute Respiratory Syndrome Coronavirus 2 Receptors in the Human Heart Using Single-Nucleus RNA Sequencing
Source: Front Cardiovasc Med. 2021 Nov 30;8:757362. doi: 10.3389/fcvm.2021.757362 (PMC8669371; doi:10.3389/fcvm.2021.757362)
Supplement: Supplementary file 2 [file Data_Sheet_2.pdf]

# Appendix A. Supplementary Data

# Detailed analyses of the expression patterns of potential SARS-CoV-2 receptors in the human heart using single-nucleus RNA sequencing

Jie Ren, MD; Yuze Zhang, BS; Shishi Liu, BS; Xiangjie Li, PhD; Xiaogang Sun, MD

**Supplementary Table 1. Basic information of human heart donors.**

| ID. | Age (years) | Gender | BMI (kg/m2) | Ethnicity | LVEF (%) |
|---|---|---|---|---|---|
| 1 | 39 | M | 21.10727 | European | 60 |
| 2 | 47 | F | 28.32658 | European | 60 |
| 3 | 51 | F | 25.59374 | European | 50 |
| 4 | 52 | F | 24.24392 | European | 75 |
| 5 | 54 | M | 20.71569 | European | 65 |
| 6 | 59 | M | 19.78997 | European | 60 |
| 7 | 60 | F | 24.60938 | European | 65 |

M= male, F= female, BMI= body mass index, LVEF= left ventricular ejection fraction

**Supplementary Table 2. the expression of accessory proteases in human heart.**

| Cell type | N_expressed_ACE2 | N_total | prop_TMPRSS2 | prop_TMPRSS4 | prop_TMPRSS11A | prop_TMPRSS11B | prop_FURIN | prop_CTSL | prop_CTSB |
|---|---|---|---|---|---|---|---|---|---|
| Fibroblast | 4219 | 83220 | 0.0021 | 0.0019 | 0.0028 | 0 | 0.0073 | 0.1346 | 0.0562 |
| Atrial Cardiomyocyte | 187 | 34051 | 0.0053 | 0 | 0.0053 | 0 | 0.0054 | 0.0749 | 0.2567 |
| Ventricular Cardiomyocyte | 1191 | 57906 | 0.0076 | 0.0050 | 0.0059 | 0.0008 | 0.0101 | 0.1360 | 0.1469 |
| Cytoplasmic Cardiomyocyte | 346 | 30765 | 0 | 0 | 0.0058 | 0 | 0.0145 | 0.0896 | 0.1040 |
| Pericyte | 1531 | 18467 | 0.0013 | 0.0013 | 0.0013 | 0 | 0.0085 | 0.0281 | 0.0261 |
| Macrophage | 350 | 17468 | 0 | 0.0029 | 0.0057 | 0 | 0.0229 | 0.2543 | 0.5543 |
| Endothelium | 563 | 27923 | 0.0071 | 0 | 0.0018 | 0 | 0.0178 | 0.1563 | 0.1599 |
| Adipocyte | 377 | 8658 | 0.0027 | 0 | 0.0080 | 0 | 0.0239 | 0.2546 | 0.2838 |
| Vascular Smooth Muscle | 72 | 5740 | 0.014 | 0 | 0 | 0 | 0.0278 | 0.1111 | 0.0972 |
| Neuronal | 32 | 1568 | 0 | 0 | 0 | 0 | 0.0313 | 0.0938 | 0.0313 |
| Lymphocyte | 13 | 1503 | 0 | 0 | 0 | 0 | 0 | 0 | 0.1546 |

**Supplementary Table 3. The expression features of BSG in human heart.**

| Cell type | Num_of_cells | Num_of_BSG_cells | Prop_of_BSG_cells |
|---|---|---|---|
| Fibroblast | 83220 | 3050 | 0.036649844 |
| Atrial Cardiomyocyte | 34051 | 4919 | 0.144459781 |
| Ventricular Cardiomyocyte | 57906 | 16240 | 0.28045453 |
| Cytoplasmic Cardiomyocyte | 30765 | 3844 | 0.12494718 |
| Pericyte | 18467 | 396 | 0.021443656 |
| Macrophage | 17468 | 712 | 0.040760247 |
| Endothelium | 27923 | 1310 | 0.04691473 |
| Adipocyte | 8658 | 749 | 0.086509587 |
| Vascular Smooth Muscle | 5740 | 156 | 0.0271777 |
| Neuronal | 1568 | 98 | 0.0625 |
| Lymphocyte | 1503 | 20 | 0.01330672 |

**Supplementary Table 4. The expression features of HSPA5 in human heart.**

| Cell type | Num_of_cells | Num_of_HSPA5_cells | Prop_of_HSPA5_cells |
|---|---|---|---|
| Fibroblast | 83220 | 2477 | 0.02976448 |
| Atrial Cardiomyocyte | 34051 | 1519 | 0.044609556 |
| Ventricular Cardiomyocyte | 57906 | 2455 | 0.042396297 |
| Cytoplasmic Cardiomyocyte | 30765 | 2605 | 0.084674143 |
| Pericyte | 18467 | 364 | 0.019710836 |
| Macrophage | 17468 | 559 | 0.032001374 |
| Endothelium | 27923 | 1049 | 0.037567597 |
| Adipocyte | 8658 | 468 | 0.054054054 |
| Vascular Smooth Muscle | 5740 | 118 | 0.020557491 |
| Neuronal | 1568 | 60 | 0.038265306 |
| Lymphocyte | 1503 | 26 | 0.017298736 |

**Supplementary Table 5. The expression features of HSPA5 in human heart.**

| Cell type | Num_of_cells | Num_of_KREMEN1_cells | Prop_of_KREMEN1_cells |
|---|---|---|---|
| Fibroblast | 83220 | 5676 | 0.068204758 |
| Atrial Cardiomyocyte | 34051 | 1538 | 0.045167543 |
| Ventricular Cardiomyocyte | 57906 | 4504 | 0.077781232 |
| Cytoplasmic Cardiomyocyte | 30765 | 667 | 0.021680481 |
| Pericyte | 18467 | 318 | 0.017219906 |
| Macrophage | 17468 | 736 | 0.042134188 |
| Endothelium | 27923 | 855 | 0.030619919 |
| Adipocyte | 8658 | 499 | 0.057634558 |
| Vascular Smooth Muscle | 5740 | 133 | 0.023170732 |
| Neuronal | 1568 | 54 | 0.034438776 |
| Lymphocyte | 1503 | 25 | 0.0166334 |
